# Eye-Tracking Data in the Exploration of Students’ Engagement with Representations in Mathematics: Areas of Interest (AOIs) as Methodological and Conceptual Challenges

**DOI:** 10.3390/jemr18060065

**Published:** 2025-11-05

**Authors:** Mahboubeh Nedaei, Roger Säljö, Shaista Kanwal, Simon Goodchild

**Affiliations:** 1 Department of Mathematical Sciences, University of Agder, 4604 Kristiansand, Norway; shaista.kanwal@uia.no; 2 Department of Education, Communication and Learning, University of Gothenburg, 405 30 Gothenburg, Sweden; roger.saljo@ped.gu.se

**Keywords:** eye-tracking, mathematical representations, mathematics learning, area of interest

## Abstract

In mathematics, and in learning mathematics, representations (texts, formulae, and figures) play a vital role. Eye-tracking is a promising approach for studying how representations are attended to in the context of mathematics learning. The focus of the research reported here is on the methodological and conceptual challenges that arise when analysing students’ engagement with different kinds of representations using such data. The study critically examines some of these issues through a case study of three engineering students engaging with an instructional document introducing double integrals. This study reports that not only the characteristics of different types of representations affect students’ engagement with areas of interests (AOIs), but also methodological decisions, such as how AOIs are defined, will be consequential for interpretations of that engagement. This shows that both technical parameters and the inherent nature of the representations themselves must be considered when defining AOIs and analysing students’ engagement with representations. The findings offer practical considerations for designing and analysing eye-tracking studies when students’ engagement with different representations is in focus.

## 1. Introduction

Mathematics as a discipline is communicated through different types of representations. In mathematics, unlike other fields (e.g., empirical sciences such as physics, biology, or geology), the only way to have access to mathematical objects is by using semiotic representations [1]. Kaput [2] argues that “representation and symbolization are the heart of the content of mathematics and are simultaneously at the heart of cognitions associated with mathematical activity” (p. 22). Thus, representations exist both in learners’ minds and in the external world, in books, instructional materials, and various instruments (e.g., navigators, measurement devices, charts, maps). Mathematical representations take different forms, including figures (e.g., pictures, charts, graphs), written symbols (e.g., numbers, equations), tables, and written language (e.g., words, statements) [3]. These representations cannot be understood in isolation; rather they interact with each other when constructing mathematical meaning [4]. For example, a specific formula or a particular graph makes sense because it is a part of a wider system of representations within which meanings can be established. In discussions about teaching and learning of mathematics, it has been argued in the literature that the combination of different representations may facilitate the understanding of mathematical ideas [5,6]. Strategic choices of representations in instructional settings may support learning. Therefore, it is important to understand how students read and engage with different types of representations in mathematics documents when attempting to learn.

However, analysing and evaluating the reading behaviours and meaning-making of individuals are challenging tasks. Nevertheless, over the years various methodological approaches have been developed in research to examine learning activities, including retrospective reporting through interviews, think-aloud protocols, etc. [7]. A promising approach to gain further insights into how students engage with mathematical representations is using eye-tracking technology [8,9,10,11]. In this technique, eye-tracking devices record an individual’s eye motion and gaze location as she/he attends to visual stimuli. It provides information about the location, timing, and duration of gaze towards a specified stimulus/area. While eye-tracking cannot reveal a person’s thoughts directly, it may offer valuable insights into cognitive processes and attentional focuses in real time [12]. A basic premise of the eye–mind hypothesis, proposed by Just and Carpenter [13], is that there is a close connection between where a person fixates and what they are actively processing. According to this hypothesis, visual attention and cognitive engagement are typically aligned. Eye-tracking technology has advanced significantly in recent years, and it is now possible to use it in a flexible and non-intrusive way in empirical research [14].

Eye-tracking is used increasingly in educational and psychological research, including in the field of mathematics education [7,11,15,16,17,18]. A key strength of eye-tracking is its ability to follow students’ attentional processes in mathematics learning activities at a micro-level as they unfold [16]. There is a growing body of research which employs eye-tracking when investigating students’ reading behaviours and engagement with different types of representations [7,11,15,19,20,21]. However, while empirical studies using eye-tracking continue to grow, fewer studies critically examine methodological assumptions, and the challenges that follow when different types of representations are attended to by learners. Representations (in this case figures, formulae, and texts) differ in nature and in how they mediate mathematical ideas [22]. Also, they are used very differently within mathematical documents. In this article, we argue that multiple factors must be considered when it comes to analysing eye movement data referring to representations in the context of mathematics learning. We claim that neglecting such methodological and conceptual specifications may result in inaccurate claims about students’ engagement and learning process and potentially lead to flawed conclusions. Engagement, in this study, refers specifically to cognitive engagement [23], which involves the mental effort and investment individuals dedicate to attempting to comprehend ideas and information. We will also discuss the implications of the findings for future studies in mathematics education that employ eye-tracking technology.

## 2. Literature Review

### 2.1. Representations, Learning, and Instruction in Mathematics

In the literature, the concept of “representation” does not have a uniform meaning or interpretation [3]. For example, Palmer [24] refers to representation as “something that stands for another” (p. 262), which means that it is a model of an entity, a thing or an idea, it represents. Duval [1] argues that “representations can also be signs and their complex associations, which are produced according to rules, and which allow the description of a system, a process, a set of phenomena” (p. 104). As we have already pointed out, representations are crucial in teaching and learning mathematics. At one level, learning how to construct, interpret, and relate different types of representational systems is what mathematics learning is about [25].

Janvier [26] classifies representations into two different forms: internal and external. Internal representations are “mental images” (p. 109), which individuals actively construct in their minds during cognitive activities [6]. External representations demonstrate mathematical relationships and ideas visually, for instance through diagrams, algebraic equations, or in some other way [27]. External representations can be categorized into various groups based on attributes, and the nature of the representation used in the teaching and learning of mathematics. Janvier [26] classified external representations into four categories including verbal descriptive, table, graph, and formula (equational). The study here considers students’ engagement with three of these types of external representations, including verbal descriptive (textual), formula, and figure.

#### 2.1.1. Textual Representations

Written and printed texts are core elements of mathematics teaching and learning and are always present during instructional activities. Textual representations are not just words and sentences but include a broad range of mathematical symbols and signs. Thus, the concept of text and textual representation should be understood in a broad sense as a fundamental element of mathematical meaning-making. In the specific context of textbooks, textual representation is the main resource for communicating what is to be learned and for formulating mathematical problems that learners engage with [1]. In this study, textual representations are explanations expressed through written modes of communication, and they include both verbal statements and mathematical symbols.

#### 2.1.2. Formula Representations

Formula/symbolic representations build on the use of symbolic notation systems, for instance, those that apply to variables. In the context of mathematics, Zhe [28] identified five types of symbolic representations, including equations, expressions, algebraic equations, algebraic expressions, and arithmetic formulae. A formula employs a procedure and a symbolic narrative, expressing a linguistically objectified schema embedded in symbols and relations between symbols [29]. The symbols used in mathematical formula/symbolic representations differ significantly from textual representations, but in the literature, there are different opinions about what this implies for cognitive activities. For instance, Mayer [30] and Schnotz and Bannert [31] do not differentiate between the cognitive processing of these two types of representations. Schnotz and Bannert [31] classify representations as “descriptive representations, which consist of symbols describing an object, such as spoken or written texts, mathematical equations, and logical expressions, and depictive representations, which consist of iconic signs such as pictures, sculptures, or physical models” (p. 143; italics in original). The positions of the authors [30,31] are grounded in the dual-coding theory [32], which posits that textual and pictorial information are processed through two distinct cognitive channels, therefore involve different cognitive processing. Based on this view, they consider textual and formula representations as the same type and assign them to the same cognitive processing channel. Ott [33], however, argues that symbolic representations such as formula and textual should be seen as two different types of representations, as they involve different cognitive processes. In this line of thinking, the meaning of the “symbol” in a formula (such as ∫, ∑, lim, etc.) may often be less familiar to students compared to the language used to convey similar concepts in verbal form in a written text [34,35]. In the present study, formula representation was operationalized as algebraic expressions and equations presented in a distinct manner (separately from the textual representations). This implies that statements in symbolic form (formulae), symbolic parts of theorems, and equations in the examples and their solutions are considered as formulae.

#### 2.1.3. Figure Representations

A figure represents information in spatial form and utilizes various iconic elements when representing something [36]. Figures can be pictures, diagrams, coordinate planes, or similar types of representations [3]. Figures are structured in holistic blocks, requiring a global reading approach rather than a sequential unpackaging as in the case of words or formulae in linear text [15]. To interpret a figure effectively, students must integrate all the elements within the figure into a comprehensible whole [37]. This difference between the sequential nature of texts, on the one hand, and images and icons, on the other, is fundamental when understanding meaning-making as Kress [38] points out. Text reading follows a “sequential logic”, i.e., readers attend to items presented in a fixed order. Attending to an image follows a “spatial” and “simultaneous logic” where all parts are combined into a whole to make sense of the representation. In this study, figure representations refer to 2D and/or 3D coordinate systems.

#### 2.1.4. Use and Processing of Multiple Representations

As we have pointed out, mathematical representations cannot be understood in isolation, and they are a part of, and belong to, a system of representations [4,27]. Mayer [39] highlights the role of multimodality and that learning from different representations can lead to better learning outcomes than learning from one representation only. Mayer and his colleagues [39], in a review of 11 experiments, found that students who received both visual and verbal input (pictures and words) presentations consistently performed better than those who received words only on transfer tests, assessing students’ ability to apply knowledge to new situations. Therefore, it is reasonable to assume that a combination of representations is sometimes useful and can contribute to a better understanding of mathematical concepts. However, despite this general assumption of the advantages of combining representations, researchers have reported that the presence of different types of representations may also bring difficulties for students depending on the nature of representations and how they are used [40,41]. Most studies have reported that textual representation is more helpful and less demanding than formulae [40,41,42]. For example, Leppink et al. [41] reported that formulae were more difficult to process than written textual representations of the same content. They developed a new 10-item psychometric instrument to measure what in the literature is referred to as cognitive load [43]. Cognitive load is defined as ‘‘a multi-dimensional construct that represents the load that performing a particular task imposes on the cognitive system of a particular learner’’ [44] (p. 122). In situations when the cognitive load becomes too high, the process of learning risks being impeded [45]. Leppink et al. [41], in an empirical study, changed the order of representations formats (first text, then formula and first formula, then text, respectively) and noticed that the cognitive load was higher when the participants (university students) worked with the formula whether or not it came before the textual representation or after it.

Other cognitive and representational factors can further influence how students process and engage with mathematical representations. Factors such as students’ prior knowledge, familiarity with specific representational, and the density or complexity of these representations will also play a role in how students engage with and attend to them [46,47,48]. For example, Huang et al. [46] highlight that when learners possess relevant prior knowledge of a representation and the corresponding mathematical domain, the cognitive effort required to comprehend it decreases. Moreover, the density of information presented in a representation also influences engagement, as a greater number of elements that must be processed simultaneously increases mental load, making engagement more effortful and requiring greater cognitive resources to process and integrate the information effectively [45].

In mathematics learning, engagement with representations can extend beyond cognitive processing. It also involves affective and metacognitive dimensions that influence how learners sustain attention, experience interest, and regulate their understanding [23]. However, in this study, engagement is operationalized through its cognitive component, defined as the mental effort invested in understanding information, which can be explored through eye-tracking methodology.

### 2.2. Eye-Tracking Methodology in Research

Eye-tracking is a methodology to document different aspects of individuals’ sensory perception in the visual domain, providing information on features of patterns of eye movements [49]. It provides information about patterns of reading, the location of the gaze, and the amount of time a person looks at a stimulus [50]. Eye movement data can provide insights into how attention is distributed and allow researchers to make inferences about participants’ engagement which may be linked to cognitive processes [51].

As a technology, eye-tracking offers several variables (e.g., average fixation duration, dwell time, number of fixations, etc.) which can be considered as indicators of cognitive processes and reading activities [52]. In research, one way to start analysing eye movements and measuring some of these variables is to define the so-called area of interest (AOI). How an AOI is defined is a critical element in the analysis of this kind of data. AOIs are (researcher) predefined areas of a stimulus, or of specific elements of a stimulus, such as a figure, or a formula, and they may also contain a combination of representations [52]. The issue of defining AOIs becomes especially interesting in the context of using different kinds of representations, since AOIs can have more than one type.

In technical terms, eye movement variables refer to different aspects of eye movement behaviours, which, in turn, relate to cognitive processes in different ways. For instance, a fixation refers to the period when the eyes remain focused on a particular area of the stimulus or an AOI. The duration of a fixation varies and often ranges from 200 to 300 ms to several seconds. But it can also be as short as 30 to 40 ms [53]. Dwell time refers to the total fixation duration one person spends within an AOI. Number of fixations refers to the total number of fixations within an AOI. Dwell time and number of fixations are indicators of the amount of attention the reader dedicates to one stimulus or an AOI [52]. An interesting conceptual problem is what this means, and how such indicators can be linked to cognitive processing. According to Holmqvist et al. [52], longer fixation durations and higher number of fixations may indicate deeper and more effortful processing of the information, but this is a hypothesis that needs further scrutiny.

Average fixation duration (AFD) refers to the total fixation duration within an AOI divided by the number of fixations in that defined AOI. AFD is taken as an indicator of a participant’s cognitive engagement with a task [54]. Therefore, a higher AFD on a stimulus may indicate a higher level of cognitive engagement with that stimulus or, alternatively, it may signal a high level of difficulty of a task [47,54]. Empirical research has shown that AFD increases as a text becomes conceptually more difficult for a given reader [55], for instance, when readers encounter words that are less frequent in the language [56].

#### Uses of Eye-Tracking in Research Involving Multiple Representations

In recent decades, mathematics education researchers have employed eye-tracking technology to explore a range of areas within mathematics learning, including the role of representations and the added value of using multimedia [11,15,19,48,57]. For example, Andrá et al. [15] examined 46 university students’ eye movement behaviours during completion of 43 multiple choice questions which consisted of different representations (text, formula, and figure). To analyse students’ eye movements, the authors included different variables, such as number of fixations, dwell time, AFD, and transitions (how often a gaze returned to an AOI which had already been visited). They reported that there is a clear difference in how students interacted with formulae and graphs. Students had longer AFDs and fewer number of fixations when attending to formulae compared to graphs. Moreover, the percentage of correct responses was lowest for formula-input stimuli, indicating that formulae posed the greatest difficulty. The authors argued that this is a consequence of the fact that a formula compresses information and is, in this sense, more dense. Spagnolo et al. [57] conducted an eye-tracker explorative study to investigate where students focus their visual attention when solving geometry tasks, especially how structural and textual aspects of mathematical problems guide reading behaviour. Their findings reveal that students often engage in selective reading, directing disproportionate attention to numeric or graphical elements while potentially overlooking parts of the mathematical text, which may influence comprehension.

While empirical research in this area is growing, only a few studies (e.g., [16,48]) point to the challenges involved in analysing eye movement behaviour when the research focus is on representations. In the next section, some of these challenges and analytical approaches related to AOIs in multi-representational contexts will be presented.

### 2.3. Challenges and Analytical Approaches When Defining AOIs for Multiple Representations in Eye-Tracking Research: The Case of Mathematics Learning

Analysing students’ eye movements across different types of representations presents several methodological challenges for researchers. One critical challenge is how to define proper AOIs when dealing with multiple representations. Eye movement variables will be affected by characteristics of the AOI, such as size and density of content. For example, the number of fixations and dwell time will increase when participants read larger AOIs [52]. Therefore, both the nature of the representation and the specific characteristics of the AOIs must be carefully considered.

**Figure 1:**
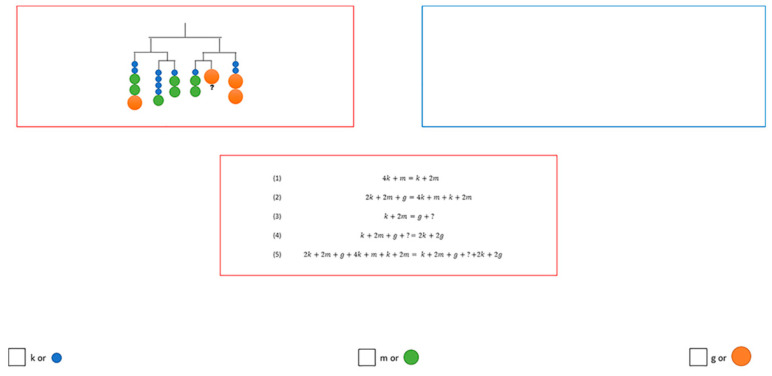
Two defined rectangular AOIs of equal size around two different stimuli (a figure and a text) used in [59] (p. 788). The red rectangular (left and lower) mark defined AOIs for the two representations, while the blue frame (upper right) indicates a third possible position for representations, which remains empty for this item. Reproduced from Malone et al. [59], “Homogeneous and Heterogeneous Multiple Representations in Equation-Solving Problems: An Eye-Tracking Study,” Journal of Computer Assisted Learning, © Authors 2020. Licensed under CC BY-NC-ND 4.0.

Many researchers have attempted to address the issue of AOIs with varying sizes by adopting different analytical approaches. Some of the previous studies attempted to avoid this issue by defining the same size of AOIs for different representations [11,58,59]. For example, Malone et al. [59] explored the influence of different representations (figure, formula, and text), as well as their combinations, on students’ problem solving of linear systems of equations. To analyse students’ eye movements and address the issue of AOIs with varying sizes, they defined distinct AOIs, each with the same fixed rectangular size (800 × 283 pixels) for the representations (e.g., a figure and a formula) (Figure 1). The authors selected several variables (e.g., mean fixation counts and mean total fixation durations) to analyse students’ eye movements when engaging with different types of representations. They reported that figures were attended mostly by students in comparison to textual and formula representations.

**Figure 2:**
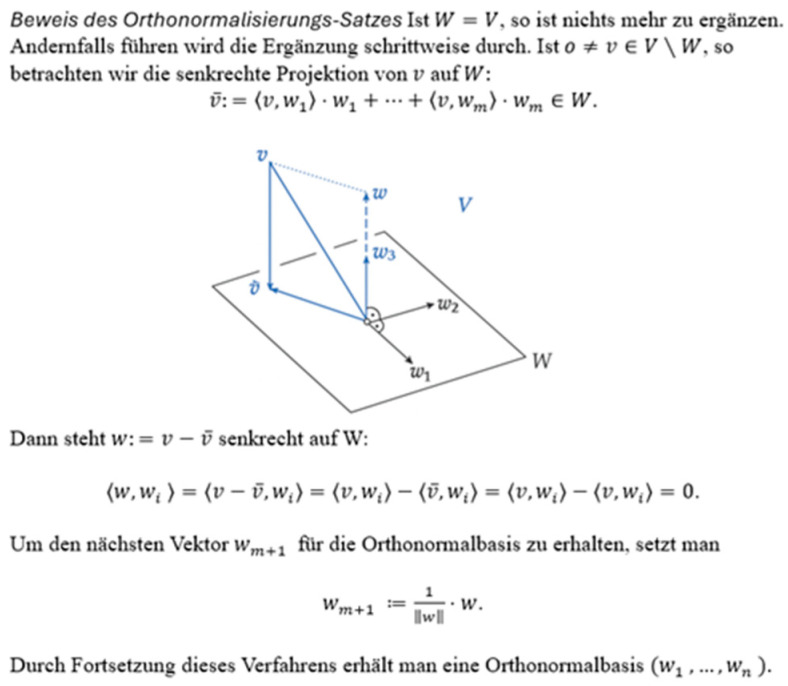
AOIs were defined to fit around the representations (the text above the picture, the picture itself, and the text below the picture), as described by the authors. Adapted from [19] (p. 124) with permission from the author.

Some studies have attempted to avoid this methodological problem of varying sizes of AOIs by standardizing them (dividing the eye movement variables of one AOI by the area of that AOI by pixel) [7,19]. For example, Beitlich et al. [19] made an in-depth analysis of the reading behaviours of eight participants as they engaged with mathematical proofs using eye-tracking. To analyse participants’ eye movements, the authors defined two AOIs: one for the texts and one for the figure, which differed in pixel size (as can be seen in Figure 2). Fixation times and fixation duration were used as primary variables. To address the problem of AOIs with unequal sizes, fixation times were standardized by dividing the total fixation time by the AOI area in pixels. They reported that only for the first item all participants showed higher fixation times on the picture, indicating greater attention to the visual representation compared to the text.

Some studies have used eye movement variables which are less sensitive to the size of AOI, such as AFD (also called mean fixation duration), revisits or time to first fixation [48,60,61]. For example, Strohmaier et al. [48] investigated students’ reading behaviours while engaging with mathematical word problems. They used AFD as one of the primary eye-tracking variables to analyse participants’ eye movements across different word problems. The authors noted that, because AFD is a standardized measure, it is less affected by variations in problem length and structure.

Some studies in the context of mathematics compare eye movement variables between different groups, such as experts versus novices [47,62]. For example, Casalvieri et al. [62] compared the eye movements of experts (PhD students, high school teachers, and mathematics graduates) and novices (first-year science students) while solving tasks on derivatives. Using fixation duration visualized through heatmaps, they found that experts adopted an analytical approach, focusing primarily on text and consulting figures for confirmation, whereas novices relied more on graphical information, often leading to misinterpretations.

Although previous research studies have employed various approaches to address the issue of varying AOI sizes in multiple representation research, few have reflected on the role of variations in representations in AOIs for students’ eye movement behaviours. Additionally, there remains a significant theoretical question regarding how to interpret observed variations in learners’ attentional patterns as they engage with different representations. This is the issue addressed in the present study.

## 3. Research Questions and Design

For this research, a case study approach was chosen to allow for an in-depth exploration of how individual students interact with different representations in a real-world learning context. In contrast to previous studies which have used a limited number of representations, this study utilizes a full document on one mathematical area containing multiple representations, as is generally the case in textbooks in mathematics when attempting to promote a deeper understanding. The area of mathematics chosen is the concept of double integrals, which is central to the mathematics curriculum for engineering students. By focusing on a small number of participants, the study aims to capture rich, detailed insights into the students’ engagement with representations and potential issues in defining AOIs that might be overlooked in large-scale studies. Specifically, this paper aims to answer the following question:

What methodological considerations should be taken into account when defining AOIs for different types of representations, and how might these considerations influence the theoretical interpretation of learners’ attentional patterns?

### 3.1. Method and Data Generation

Three first-year engineering students from a university in Norway were recruited to participate in this study. The small number of participants was a deliberate choice, as the aim of the study was exploratory and focused on conducting an in-depth analysis of individual attentional processes in response to different kinds of representations rather than achieving broad generalization. Thus, in this study we are interested in exploring the functional relationships at the inter-individual and intra-individual level, respectively, between representations and eye movements in relation to the complexity of the AOIs. Students were recruited on a priori criteria; for example, they had to take part in specific mathematics courses as part of their curriculum. Thus, they would eventually encounter the concept of double integrals in their future studies.

A brief document, intended to provide an initial introduction to double integrals, was designed by the first author. The document was based on the presentation in several calculus textbooks (e.g., [63,64]). Students had not learned about nor encountered double integrals in their courses up to that point (Before the learning intervention, the author asked students about whether they have learned double integrals before and confirmed that this was the first time they had encountered the topic. In this way, the study accounted for the effect of students’ prior knowledge of the concept), although they had some courses that addressed the basic knowledge for learning this topic, for example definite integral and its applications. The document designed as learning material includes a short introduction of double integrals, a definition of double integrals, one theorem (Fubini’s theorem), and two simple examples and their solutions. The designed document was prepared in Portable Document Format (pdf) and displayed on a computer screen equipped to track students’ eye movements when reading. The document consists of six pages. Students were able to scroll through the document and move backward and forward at a pace of their own choosing. After reading the document, students were asked to take a test designed by the first author based on the content of double integrals. In the present paper, only the data generated through eye-tracking will be reported, as the focus of this paper is on the methodological aspects of using eye-tracking when studying different representations.

The equipment used in this study was a screen-based eye-tracker, which was placed below the screen and which recorded students’ eye movements from a distance. Students’ eye movements were captured using Tobii Pro Nano (this product has been replaced by Tobii Pro Spark: “https://www.tobii.com/products/eye-trackers/screen-based/tobii-pro-nano (accessed on 22 October 2025)”) eye-tracker, set to sample at 60 Hz. The Tobii Pro Nano is a remote eye-tracker equipped with one binocular camera, and it typically achieves eye-tracking accuracy of 0.3°. Participants viewed stimuli presented on a screen from a distance of approximately 65 cm, without any head restriction. For each participant, the eye-tracker was calibrated using a 9-point display. Each participant took part individually in a quiet room at the university. The first author was present in the room to ensure the proper functioning of the eye-tracking equipment and to address any potential needs or issues that students might have during learning sessions. However, she refrained from disturbing students during learning sessions. There were no time constraints for students, and they could read the material at their own preferred pace. The duration for engaging with the content was different for students. Student 1 read the content for 15 min, Student 2 used 22 min, and for Student 3 it took 30 min before he stopped reading. The first author explained the project to the students before reading and informed them that they would be asked to complete a written test on the content and their understanding afterwards. Students did not have the possibility to take notes while reading the document because the screen calibration would be lost in that condition, and the data would be affected. Moreover, students were informed that their eye movement would be tracked during learning sessions.

To foster a better understanding of double integrals for students, the designed document was written in an introductory textbook style, and it included different types of mathematical representations such as formulae, texts, and figures. The content is structured in a way that establishes connections between the information presented in the texts, formulae, and figures. This integrated approach is designed to offer students an authentic and comprehensive view of the topic, facilitating a better understanding of the concept presented in the document. Table 1 provides definitions of each representation type used in this study, along with an example for each.

Two senior lecturers in mathematics and three senior lecturers in mathematics education examined the structure of the double integrals document. The lecturers reviewed the document to check the coherence of the material and to ensure that the information presented was appropriate for the students. Additionally, a pilot study was conducted with two students before the main study to examine the designed document and to check the proper work of eye-tracking technology. A few changes were made after the pilot, such as adding extra examples to the document to increase the clarity of the topic for students.

### 3.2. Data Analysis

A series of steps was undertaken to analyse students’ eye movements as they engaged with different types of representations in the double integrals document. First, different representations (texts, formulae, and figures) within the designed document were identified and categorized according to the definitions outlined in Table 1. Next, in order to analyse students’ eye movement within each representation and calculate eye movement variables, distinct AOIs needed to be defined and drawn for each representation in the document. However, as highlighted in the literature, several methodological approaches can be taken for defining AOIs. We considered a selection of these approaches (e.g., defining AOIs with same size and standardization) to analyse students’ engagement with representations; however, each approach introduced potential issues to our data analysis.

#### Methodological Considerations in Defining AOIs for Different Representations and Analysing Eye Movement Behaviour

**Figure 3:**
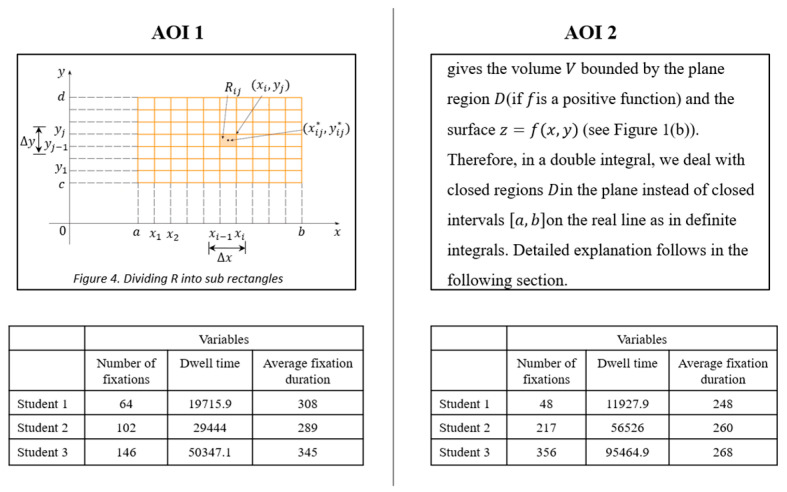
Two defined AOIs of equal size (one figure and one text), along with students’ eye movement behaviour when reading them.

First, we considered that defining AOIs of same sizes across the three representations was not feasible and it could lead to inaccurate analysis and results. For instance, Figure 3 illustrates two different representations (a figure and a text) with equal size positioned in the double integrals document. Although the size of two AOIs (the rectangular shape around the figure and text, each 101,865 pixels) was the same, the figure contains fewer characters compared to the text. As shown in the tables below the figures, students 2 and 3 had a higher number of fixations and dwell time on the text, which is reasonable because the text contains more characters to process than the figure. Therefore, relying solely on spatially defined AOIs without considering content density can lead to questionable interpretations of attentional distribution. In this case, longer viewing times might be misinterpreted as greater engagement with the text, when in fact the text simply has more elements to read compared to the figure.

**Figure 4:**
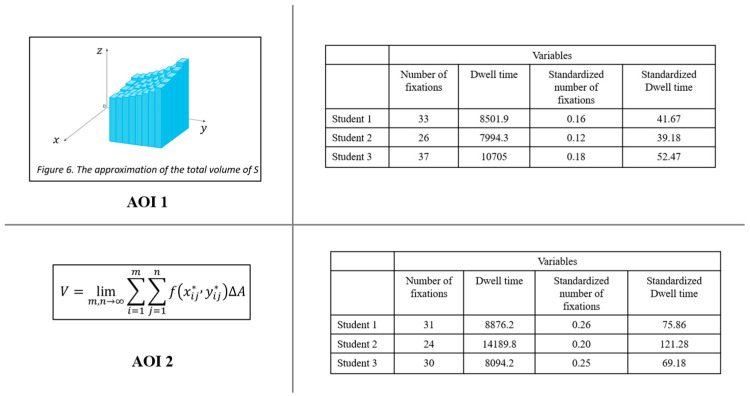
Two defined AOIs (a figure and a formula) of different sizes, along with students’ dwell time and number of fixations during reading them.

Moreover, standardizing AOIs with different sizes for different types of representations (formula, figure, and text) was not feasible either. Figure 4 shows two different AOIs (a formula and a figure positioned in a rectangular shape) from the double integrals document. As can be seen, the two AOIs are different in size, and the figure consists of a significant proportion of white space which normally is not attended to by readers. In contrast, the formula contains less proportion of white space, and its AOI was drawn tightly around it. When one standardizes these AOIs, one includes the white space in the figure, which students did not look at. This would lead to a miscalculation and a lower density value for the figure compared to the formula. For example, Student 1 spent 8501.1 ms on the figure and 8876 ms on the formula. When standardized by pixel size, these values became 41.67 ms per pixel for the figure and 75.86 ms per pixel for the formula. However, the figure’s value was calculated using the entire AOI area, including substantial white space that learners typically do not fixate on. This artificially lowers the standardized dwell time for the figure, despite similar overall attention, leading to misinterpretations of attention and cognitive effort across different representation types. In this case, the lower standardized dwell time for the figure could be mistakenly interpreted as indicating significantly less cognitive effort and engagement, when in fact it reflects a methodological artifact caused by including irrelevant white space in the AOI calculation.

**Figure 5:**
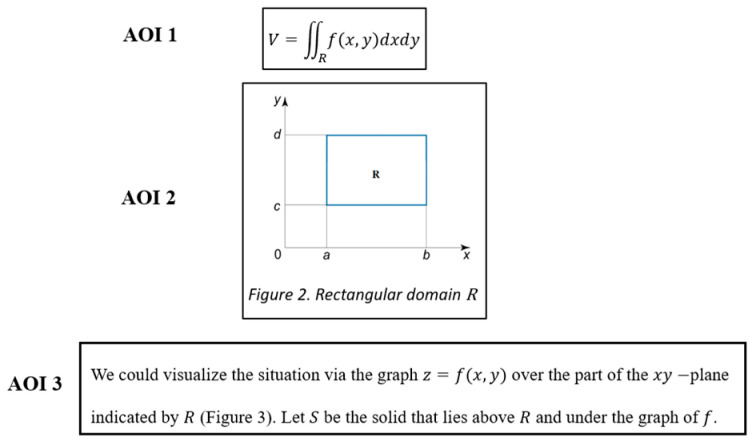
Example of AOIs defined for a formula (AOI 1), figure (AOI 2), and text (AOI 3) within double integrals document, each fitted around its respective representation.

Based on the considerations discussed for defining AOIs, for this study, we defined and framed each AOI around texts, formulae, and figures separately (see Figure 5, which shows how AOIs were defined for a part of the double integrals document).

The size of each AOI varied, and the complete document comprised 40 AOIs, consisting of 20 texts, 13 formulae, and 7 figures. The raw data of the number of fixations and dwell time for each AOI were considered inappropriate measures due to variations in size, the varied nature of representations, and the number of AOIs. Further, calculating these values in proportion to the size of each AOI is inappropriate due to the varying nature of the content of the AOIs, and how space is used within each AOI. Thus, the AFD for each type of representation was considered and calculated. This variable minimizes the influence of AOI size, layout, and visual density. AFD, by averaging the duration of each fixation regardless of AOI size, offers a stable basis for comparison across differently sized and structured visual elements, making it technically better suited for analysing eye-tracking data where AOIs are not uniform.

## 4. Results

**Figure 6:**
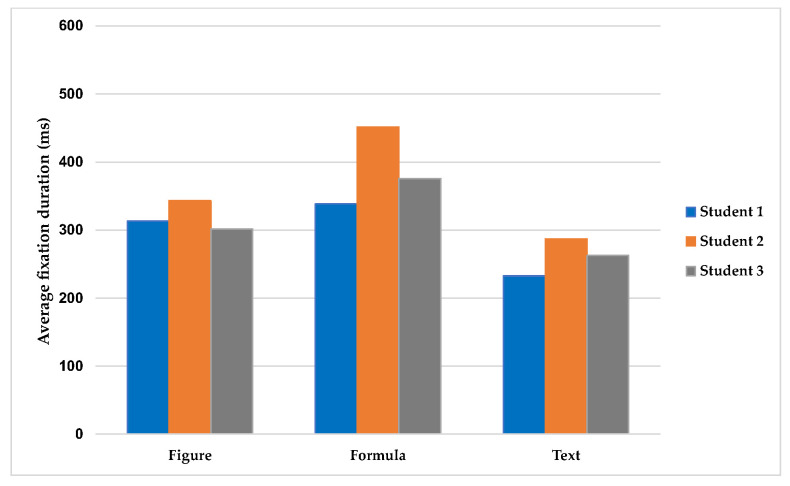
Students’ AFD for each type of representation.

Using students’ AFDs as a measure reveals that students had longer AFD when interacting with formula representations, followed by figure representations, and the lowest with textual representations. Figure 6 presents three students’ AFD for each type of representation.

As can be seen in Figure 6, there are clear differences in how students engaged with various types of representations, both between different students (inter-individual) and within individual students (intra-individual). At the inter-individual level, each student engaged differently with the three representations, as indicated by their varying AFD for each representation. However, these differences make a consistent pattern across all three students in terms of notably longer AFD on formula representations compared to figure and textual representations, indicating greater cognitive effort and engagement with formulae. Figure representations received moderate and relatively consistent AFD among the students, while textual representations had the lowest AFD overall. At the intra-individual level, patterns of engagement also varied within each student. For example, Student 2’s AFD differed considerably across representation types, reflecting greater variability in attention allocation. Student 1 showed smaller differences in AFD, particularly between figures and formulae. These patterns of eye movement reveal both common and unique strategies students use to process different types of representations.

One-way Analysis of Variance (ANOVA) was used to explore whether the differences noticed were significant, and, if so, to obtain estimates of the effect size (While statistical inference is limited due to the small sample size, ANOVA and pairwise comparison techniques were employed in an exploratory manner. Their role here is not to produce significant results, but to serve as indicators of variation between individual students. This quantitative lens supplements the case study by highlighting micro-level differences that might inform or anticipate patterns in larger-scale research, where these methods are more commonly applied to large datasets). Results from the ANOVA are presented in Table 2.

Based on the ANOVA results, the analyses suggested differences in AFD between the three representations (text, formula, and figure) for all three students. The effect size values indicate substantial variation in how long, on average, each student fixated on the different types of representations. Subsequently, a post hoc test was undertaken to conduct pairwise comparisons between the representations to explore which representation contributed most to the observed differences. As shown in Table 3, formulae tended to differ from the other types of representations for all three students, with some variation across individuals. For Student 1, the comparisons suggested differences in AFD between formula and text as well as between figure and text, indicating that this student’s eye movements varied when engaging with formulae and figures compared to texts. For students 2 and 3, formulae appeared to differ from both texts and figures, suggesting that their fixation behaviours were distinct when reading formulae relative to the other representation types.

Overall, the results show that students’ patterns of engagement varied when reading the three types of representations. This variation was observed both between students and within each individual, reflecting a nuanced interaction shaped by the representational forms encountered. The finding suggests that representations are not interpreted uniformly; rather, the cognitive work involved varies depending on the type of representation from student to student and within individuals. Therefore, this shows that depending on which representations are included in an AOI, students’ patterns of eye movement will be different, underscoring the need to consider the representational characteristics of each AOI during analysis.

## 5. Discussion and Conclusions

This study explores key methodological considerations in defining AOIs when different types of representations are at the focus, and how these considerations can influence the theoretical interpretation of students’ attentional patterns. In this exploratory study, the eye movements of three engineering students are examined when they engage with a full mathematics document on the topic of double integrals that integrated text, figure, and formula representations, making it closer to real learning materials such as textbooks. To analyse students’ eye movements, AOIs were defined around these representations, resulting in 40 distinct AOIs (20 texts, 13 formulae, and 7 figures). The variation in size, number of AOIs, and the nature of representations included in these AOIs introduced challenges in the analysis, which led us to propose methodological and conceptual considerations to inform future research.

The first consideration concerns defining and interpreting AOIs in multi-representational contexts. That is, the specific type of representations included within an AOI can influence the interpretations of students’ eye movements and reading behaviours. In other words, the characteristics of representations can shape eye movement patterns, meaning eye movement data from two AOIs containing different types of representation cannot be interpreted in the same way. As shown by the findings of this study, these three students showed longer AFD on formulae compared to figure and textual representations. The longer AFD on formula representations could be attributed to the challenging nature of the formula leading to an increase in the cognitive load, as highlighted by previous research (e.g., [15,48]). The mathematical formulae are packed with many symbols and notations, which makes it difficult for learners to comprehend and requires more time to process all the elements in the formula, thereby increasing cognitive load [35,41]. Therefore, unpacking the information presented in a formula requires more cognitive processing for learners, the longer AFD for the formulae is thus interpreted as deeper engagement (higher mental effort) with this type of representation compared to the other two as reported by previous research such as Andrá et al. [15]. Moreover, the result of this study suggests that depending upon the nature of the representation, students’ eye movement behaviours can vary even within the individuals. For example, Student 1 engaged more with formula and figure representations compared to texts, resulting in higher AFD for these two types. This difference in Student 1’s different engagement with the three representations could have arisen due to additional factors such as his individual style of reading, the learned manner of reading mathematical documents, the place of the representation (AOI) within the document, etc., factors which are beyond the scope of this study. The key point here is that these cases illustrate that students can engage with representations in non-uniform and context-sensitive ways, which suggests that the design and interpretation of AOIs cannot be uniform across all representation types, as each representation affords different modes of engagement and cognitive demand. Therefore, it is important to consider the specific functions and nature of different representations when defining AOIs, acknowledging that these differences might shape students’ engagement with the material. This challenge becomes particularly significant in authentic instructional materials, such as the mathematical texts used in this study, where multiple representations interact to convey meaning. Thus, defining AOIs in multi-representational contexts is not just a technical decision, but a theoretical and conceptual challenge that requires further investigation.

The second consideration that this study suggests is that the ways of, and approaches to, defining AOIs for different types of representations significantly affect both the outcomes and the interpretation of students’ eye movement behaviours. While some prior studies have proposed solutions for defining AOIs (see [7,48,59] above) and analysing eye movement behaviours in multi-representational contexts, this study demonstrates that such approaches can still lead to misleading results. Specifically, this study shows that using fixed-size AOIs to compare visual attention across inherently different representations will often be misleading. For example, a textual representation might contain more characters than a figure, leading to potentially higher number of fixations and dwell time for text compared to the figure. Although some prior studies have attempted to control AOI size using fixed AOIs (e.g., [58,59]), these approaches may not adequately account for the density of visual representations, as shown in our analysis. Moreover, this study highlights the limitations of the methodological strategy of standardizing eye movement variables such as dwell time and number of fixations by dividing them by the pixel size of AOIs, as seen in studies like [7,19]. While this method aims to account for differences in AOI size, it becomes problematic when comparing different types of representations. This is primarily due to the varying proportions of white space across representations. For instance, figures often contain substantial white space that is not typically fixated on by learners, whereas AOIs for formulae or texts are densely packed with characters and tightly bounded. Consequently, students may appear to spend more time on texts or formulae simply because the larger size of figure AOIs reduces the relative values when standardized, not necessarily because they require more cognitive effort.

In addition to the size of AOIs, factors such as the complexity of information within representations, modality, and familiarity with representations should be considered when analysing students’ learning behaviour in AOIs. Complexity of the information of each representation has importance when attempting to understand how students process mathematical representations while learning. The presence of different representations and the content within AOIs are consequential for the complexity in terms of the cognitive demands for the learners [40]. Moreover, modality can shape how learners allocate attention and process information, as different modes engage distinct perceptual and cognitive channels [30,31]. Text and formula representations demand sequential reading, whereas figures afford more global visual processing, aligning with dual coding and multimedia learning perspectives [32,39]. Differences can also occur within the verbal modality itself, for example, between formula and text representations, as emphasized by Ott [33]. Familiarity and previous knowledge also influences students’ engagement with representations: familiar representations allow smoother processing and more efficient interaction, whereas unfamiliar ones demand greater effort and sustained attention to understand the information presented [43,46,47]. Moreover, in this study, engagement was operationalized primarily in cognitive terms. However, engagement is a multi-dimensional construct that also includes affective (interest, motivation) and metacognitive (self-monitoring, strategy use) dimensions [23]. Therefore, analysing students’ engagement with representations goes beyond simply defining AOIs, and researchers should take different dimensions into account to gain a more comprehensive understanding of students’ engagement with mathematical representations.

Another important point to consider is how data aggregation affects interpretation of eye-tracking data. Our findings show that these three students interact with mathematical representations in distinct ways. Within-student analyses showed clear differences (especially between formula and textual representations), but these differences diminished when data were aggregated across three students. This reflects a common issue in large-sample studies, where averaging can obscure meaningful individual variation. Thus, we suggest that large-scale studies should be accompanied by in-depth analysis and case study based designs if the goal is to make sense of students’ engagement and cognitive activities (e.g., [17]).

Eye-tracking data provide rich insights into students’ attention and interaction with mathematical presentations [11,16]; however, dilemmas in analysis of eye movement behaviours arise when multiple types of representations are involved. This case study is intended as a methodological contribution and basis for future large-scale investigations in mathematics education using eye-tracking. Future studies in mathematics education should therefore (1) make AOI design decisions explicit, (2) carefully consider how AOIs are constructed when different representations are involved, (3) consider the characteristics of different representations when analysing students’ engagement and learning process, and (4) recognize that other contextual and individual factors may also play a role in students’ engagement. AOI design choices influence what we see and understand about students’ engagement. Neglecting such details can lead to misleading interpretations of students’ engagement and learning process. This study contributes to a growing body of work aimed at refining the tools and techniques used to investigate learning in mathematics. As eye-tracking becomes more accessible in mathematics educational research, the field must continue to refine its methodological standards to ensure that the data collected lead to meaningful and accurate insights into how mathematics is taught and learned.

## Figures and Tables

**Table 1:** Definition and an example of each representation used in the designed double integrals document.

Representation	Definition of Each Representation	Example
Textual	Textual representations are explanations expressed through written statements, and they include both verbal statements and mathematical symbols to provide a clear explanation for the students. The simple equations and symbolic expressions within the textual representations complement the explanations provided in the written text. They do not introduce new or complex symbolic relationships.	A double integral gives the volume V bounded by the plane region *D* (if *f* is a positive function) and the surface z = f(x,y). Therefore, in a double integral, we deal with closed regions *D* in the plane instead of closed intervals [a,b] on the real line as in definite integrals. Detailed explanation follows in the following section.
Formula	Formula representations are algebraic expressions and equations that were presented in a distinct manner (separately from the textual representations) such as statements in symbolic form (formulae), symbolic parts of theorems, and equations in the examples and their solutions. Formula representations, in this study, condense the information in a shorter way with using only mathematical symbols and equations.	$V=\iint_{D}f\left(x,y\right)dxdy$
Figure	Figure representations refer to 2D and/or 3D coordinate systems in this study.	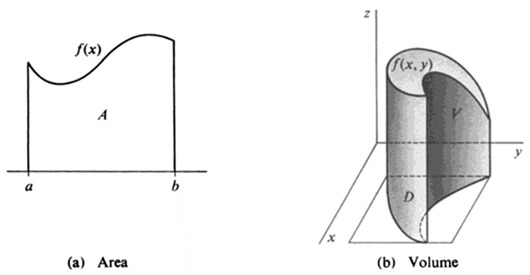

**Table 2:** ANOVA statistics for three students.

	Student 1	Student 2	Student 3
F, df	28.23, 2	17.15, 2	12.76, 2
Significance *p*	<0.001	<0.001	<0.001
Effect size $\eta_{\rho }^{2}$	0.6	0.48	0.41

**Table 3:** Multiple comparison between three representations using post hoc test.

Compared Representation	Student 1			Student 2			Student 3		
	Mean	S.E.	*p*	Mean	S.E.	*p*	Mean	S.E.	*p*
Formula vs. Text	105.6 *	16.5	<0.001	165.2 *	29.17	<0.001	113.2 *	27.9	<0.001
Figure vs. Text	80.5 *	17.23	<0.001	56.27	43.27	0.25	38.9	34.4	0.347
Formula vs. Figure	25.01	21.9	0.4	108.9 *	49.93	0.016	74.3 *	36.7	0.042

* The mean difference is significant at the 0.05 level. The mean difference here refers to the difference in the means (average values) of AFD between specific type of representations. The mean difference tells how much, on average, AFD in one type of representation differs from another one.

## Data Availability

The original contributions presented in this study are included in the article. Further inquiries can be directed to the corresponding author.

## Abbreviations

The following abbreviations are used in this manuscript:

AOI	Area of Interest
AFD	Average Fixation Duration
ANOVA	Analysis of Variance
